# Posterior Reversible Encephalopathy Syndrome in a Patient With SARS-CoV-2 Infection Treated With Tocilizumab

**DOI:** 10.7759/cureus.13475

**Published:** 2021-02-21

**Authors:** Krishna Talluri, Naveena Lall, Marcos A Moreno, Laura Nichols, Dinesh Bande

**Affiliations:** 1 Internal Medicine, Sanford Health, Fargo, USA; 2 Internal Medicine, University of North Dakota School of Medicine and Health Sciences, Grand Forks, USA; 3 Internal Medicine, University of North Dakota School of Medicine and Health Sciences, Fargo, USA

**Keywords:** case report, sars-cov-2, covid-19, tocilizumab, pres

## Abstract

As the world has struggled to adapt to the coronavirus disease (COVID-19) pandemic, new evidence has emerged suggesting that severe acute respiratory syndrome coronavirus 2 (SARS-CoV-2) infection may manifest with a wide variety of neurologic symptoms. We present the case of a 70-year-old patient hospitalized for COVID-19 related pneumonia who was treated with off-label interleukin (IL)-6 inhibitor tocilizumab and eventually developed prolonged delirium. MRI findings were consistent with posterior reversible encephalopathy syndrome (PRES). PRES was felt to be from SARS-CoV-2 infection, tocilizumab, or a combination. The patient received symptomatic treatment without success. These findings are consistent with few other recent reports, which have chronicled PRES findings in patients with SARS-CoV-2 infections. However, this is only the second example of PRES in a COVID-19 patient treated with tocilizumab. While cases of PRES have been noted to occur with other infectious diseases, clinicians should be aware of the association with SARS-CoV-2 infection and tocilizumab therapy, particularly when considering tocilizumab treatment outside its approved indication. Future research efforts are needed to establish evidence-based guidelines for the management of these patients.

## Introduction

Beginning December 2019 in Wuhan China, severe acute respiratory syndrome coronavirus 2 (SARS-CoV-2) was identified as the pathogen responsible for causing cases of coronavirus disease (COVID-19). Initial reports of COVID-19 described patients as presenting with malaise, fever, and sore throat; but in the most severe cases, pneumonia, respiratory distress, and other organ involvement were also noted [1-2]. Recent literature has suggested that up to one-third of patients with SARS-CoV-2 infection have some evidence of neurologic involvement. Neurologic manifestations have included severe headaches, visual disturbances, olfactory disturbances, stroke, seizures, and encephalopathy [3-4]. In our case report below, we describe a 70-year-old gentleman who presented with COVID-19 pneumonia and later progressed to have neurological symptoms of encephalopathy with imaging findings consistent with posterior reversible encephalopathy syndrome (PRES) after treatment with tocilizumab, an interleukin (IL)-6 inhibitor.

## Case presentation

A 70-year-old gentleman with a history of mild persistent asthma, hypertension, and coronary artery disease presented to our hospital with a five-day history of fever, cough, and exertional dyspnea. Upon admission, he was afebrile at 98.5 ^o^F with a blood pressure of 124/72 mmHg with oxygen saturation of 92% on room air. On exam, he was alert and oriented with unremarkable neurological findings. Bilateral rales were noted on pulmonary auscultation. CT chest revealed patchy ground-glass opacities in all lobes, along with bronchiectasis in bilateral posterior lower lobes. White blood cell count (WBC) was 5.5 K/ul (normal 4-11 K/ul), lactate dehydrogenase 422 U/L (normal 125-245 U/L), aspartate aminotransferase 66 U/L (normal 0-35 U/L) and alanine aminotransferase was 71 U/L (normal 0-55 U/L). The rest of the liver function test and renal profile were within normal limits. Nasopharyngeal swab SARS-CoV-2 polymerase chain reaction (PCR) test was positive (Cepheid GeneXpert, Sunnyvale, California). He was started on azithromycin and hydroxychloroquine per our institutional standardized treatment plan for COVID-19 patients at the time. Within 24 hours of hospitalization, his respiratory status deteriorated, and he was intubated.

The patient showed signs of hyperactive delirium during a sedation vacation on day five of hospitalization, which continued through the rest of his stay. He ultimately required symptomatic treatment with multiple medications, including quetiapine, dexmedetomidine, valproate sodium, and haloperidol. IL-6 level was elevated at 118 pg/ml (normal < 1.8 pg/ml), and the patient was treated with two doses of tocilizumab on days six and 13 as off-label use. On day 16, sporadic episodes of elevated blood pressures up to 160/100 mmHg were first noted, and his home antihypertensive medications were restarted. His respiratory status gradually improved, and he was extubated on day 17. However, delirium continued post-extubation, and it was unresponsive to symptomatic treatment. He was discharged to long-term acute care (LTAC) facility on day 19 for further care, following two consecutive negative SARS-CoV-2 PCR tests.

**Figure 1:**
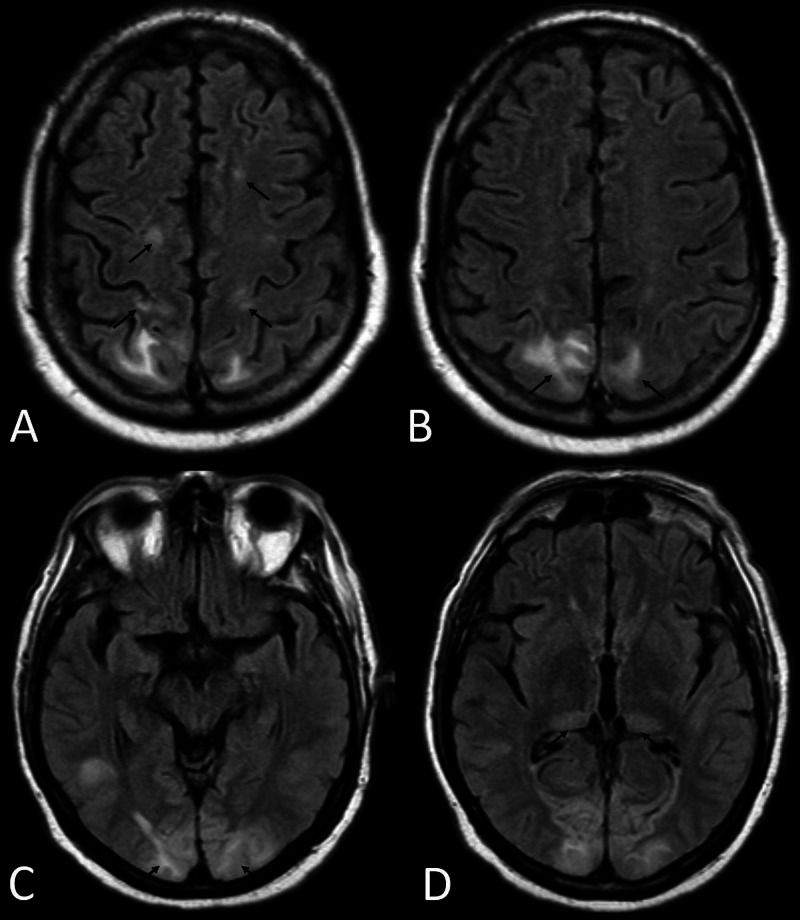
Images A-D demonstrate abnormal symmetric cortical and subcortical white matter FLAIR signal hyperintensity involving bilateral frontotemporal lobes (Image A), occipital lobes (Images B and C), and posterior thalami (Image D) on MRI

**Figure 2:**
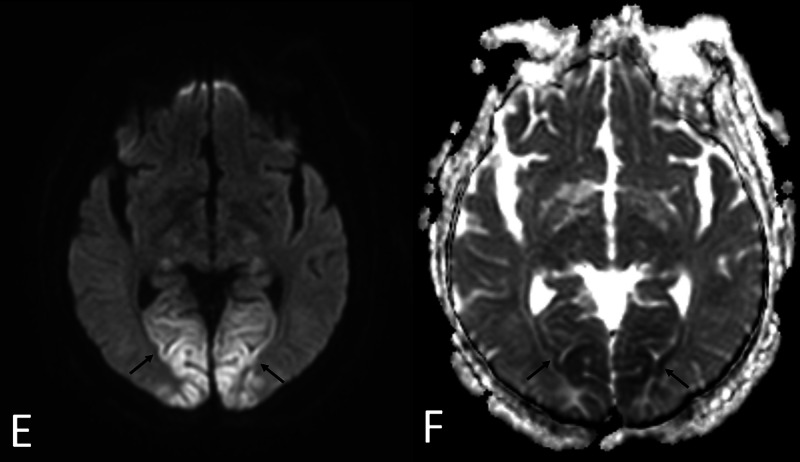
Images E-F show restricted diffusion in a similar distribution concerning for complicated PRES

While at the LTAC, the patient progressed to hypoactive delirium. Systolic blood pressure ranged from 140-160 mmHg, and diastolic blood pressure ranged from 90-100 mmHg. Workup for infection and electrolyte abnormalities was insignificant. Head CT was suspicious of PRES due to findings of fairly symmetric abnormal low attenuation seen in the bilateral posterior occipital lobes involving the subcortical white matter and potentially involving the cortex. Further evaluation with MRI showed abnormal restricted diffusion again in a symmetric pattern involving bilateral occipital lobes, bilateral posterior thalami and left temporal lobe. In addition, there was abnormal cortical and subcortical white matter fluid-attenuated inversion recovery (FLAIR) signal hyperintensity involving both bilateral occipital lobes as well as the frontoparietal lobes paramidline in distribution. The overall fairly symmetric pattern suggested posterior reversible encephalopathy syndrome (Figures 1, 2). Cerebrospinal fluid (CSF) analysis revealed normal parameters including WBC count 4/uL (normal 0-5 /uL). Meningitis and encephalitis PCR panel was negative for infection. Electroencephalogram (EEG) was negative for seizure activity. The patient’s presentation was thought to be consistent with PRES due to SARS-CoV-2 infection versus treatment with tocilizumab or a combination. Intermittent elevation in blood pressure that started much later in his hospital course was felt to be a confounder. By day 25, he was febrile, tachycardic, hypotensive, and hypoxic. Workup revealed leukocytosis, elevated creatinine, negative SARS-CoV-2 PCR test and multifocal infiltrates on chest x-ray, likely secondary to aspiration pneumonia. He was diagnosed with sepsis with impending multi-organ failure, and he passed away on day 27 following initiation of comfort cares.

## Discussion

Coronavirus Disease 2019, has shown to be associated with numerous neurological manifestations. However, the radiologic and clinical manifestation of PRES has been a rarely reported condition in these patients. Literature is evolving on the topic of encephalopathy, and to date, only a few studies have reported PRES-related MRI findings. Post-mortem MRIs of 19 patients in Belgium showed PRES in one patient [5]. Of a review of another set of MRIs of 20 patients in Italy, one patient showed PRES [6]. Only a few more reports of COVID-19 patients were reported with similar MRI findings [7-9]. Patients with PRES may present with headaches, mental status or visual changes, or even seizures [10]. Pathogenesis remains unclear, but one of the common theories includes endothelial dysfunction, as seen in severe inflammatory states such as sepsis or cytotoxic therapies [11].

Here, we present another case of COVID-19 encephalopathy manifesting as delirium with MRI finding of PRES. Unique about our patient is that he received tocilizumab for severe COVID-19 infection prior to the diagnosis of PRES. Elevated pro-inflammatory markers including IL-6 are associated with increased mortality in COVID-19 cases and blocking this inflammatory pathway has been studied as a treatment modality. Administration of this IL-6 inhibitor tocilizumab has been shown to result in improvement of clinical and inflammatory markers within 24 hours of infusion among patients with COVID-19 [12]. However, a rare yet potential complication of this immunomodulating drug may be PRES as well. A tocilizumab-associated PRES has been reported in a patient with juvenile idiopathic arthritis [13].

Both COVID-19, in a setting of uncontrolled immune response, and cytotoxic medication such as tocilizumab used in the management of severe COVID-19, may lead to endothelial dysfunction and potentially subsequent PRES. In our patient, the risk of PRES from COVID-19 may have been augmented by concomitant administration of this immunomodulatory tocilizumab. With the increase in the use of tocilizumab, either investigational or off-label use in severe COVID-19 patients, this potential complication will need to be recognized and considered in patients complaining of neurologic symptoms.

## Conclusions

Clinicians should consider PRES in their differential while managing COVID-19 patients with encephalopathy, especially those who receive immunotherapy like tocilizumab. Work-up for PRES should be undertaken early-on in these patients, as early diagnosis may guide management and improve outcomes. Further data and research are necessary to establish diagnostic tests, treatment options, and outcomes.
